# Age-Infusion Approach to Derive Injury Risk Curves for Dummies from Human Cadaver Tests

**DOI:** 10.3389/fbioe.2015.00196

**Published:** 2015-12-14

**Authors:** Narayan Yoganandan, Anjishnu Banerjee, Frank A. Pintar

**Affiliations:** ^1^Department of Neurosurgery, Medical College of Wisconsin, Milwaukee, WI, USA; ^2^Division of Biostatistics, Medical College of Wisconsin, Milwaukee, WI, USA

**Keywords:** biomechanics, injury risk curves, statistical analysis, logistic regression, impact loading, probability curves, matched-pair tests

## Abstract

Injury criteria and risk curves are needed for anthropomorphic test devices (dummies) to assess injuries for improving human safety. The present state of knowledge is based on using injury outcomes and biomechanical metrics from post-mortem human subject (PMHS) and mechanical records from dummy tests. Data from these models are combined to develop dummy injury assessment risk curves (IARCs)/dummy injury assessment risk values (IARVs). This simple substitution approach involves duplicating dummy metrics for PMHS tested under similar conditions and pairing with PMHS injury outcomes. It does not directly account for the age of each specimen tested in the PMHS group. Current substitution methods for injury risk assessments use age as a covariate and dummy metrics (e.g., accelerations) are not modified so that age can be directly included in the model. The age-infusion methodology presented in this perspective article accommodates for an annual rate factor that modifies the dummy injury risk assessment responses to account for the age of the PMHS that the injury data were based on. The annual rate factor is determined using human injury risk curves. The dummy metrics are modulated based on individual PMHS age and rate factor, thus “infusing” age into the dummy data. Using PMHS injuries and accelerations from side-impact experiments, matched-pair dummy tests, and logistic regression techniques, the methodology demonstrates the process of age-infusion to derive the IARCs and IARVs.

## Current Knowledge and Advances in Dummy-Based Injury Assessments

Anthropomorphic test devices (ATDs) referred to as the dummy in the automotive and manikin in the military fields are important biomaterials, albeit composite, used to advance human safety (Backaitis and Mertz, 1994). In the field of mechanics of biomaterials, determinations of injury criteria are critical to develop dummies with biofidelity to assess and mitigate injuries in environments, such as motor vehicle crashes, military events, and sports activities (Yoganandan et al., 2014b). The mechanics of the physical device (dummy) and the biomechanics of the human body and or body region are interrelated. From an automotive perspective, experiments using post-mortem human subjects (PMHSs) are used to reproduce field injuries, obtain biomechanical metrics, and correlate the two variables to achieve this goal (Yoganandan et al., 2007, 2011). For example, the development of the human head injury tolerance criterion was based on this method (FMVSS-208, 2001). Extra-cranial accelerations from drop tests of isolated PMHS heads and intact PMHS combined with skull fracture pathologies were used in the promulgation of the head injury criterion (HIC) (Evans et al., 1958; Lissner et al., 1960). This index continues to be used for different impact modes. The use of this criterion has been responsible for mitigating skull fractures and brain injuries in automotive and other environments (Yoganandan et al., 2015b). The HIC used in the federalized Hybrid III test device for evaluating vehicle crashworthiness was directly based on PMHS accelerations. An acceleration-based criterion is much more dependent on the attached mass because the head mass of the Hybrid III device is the same as the mass of the human head by design; hence, HIC applies to both surrogates. Other factors, such as age and gender, do not influence this parameter. However, this is not true for all regions of the human body. Therefore, a different process, matched-pair design, is used for developing injury criteria for other regions.

### Matched-Pair Test Design Used in Current Injury Criteria

The underlying concept in the matched-pair test design is to conduct experiments under differing sets of initial conditions using both the biological surrogate and the physical device. For the same initial condition, often delivered as a pulse in terms of change in velocity of the sled based on its acceleration–time signal and end/boundary conditions in each test, the biological surrogate produces differing outcomes measured in the form of mechanical metrics, such as spine acceleration. The surrogate also produces differing anatomical/structural outcomes, identified or inferred in terms of injuries. For example, fracture in one specimen test and no fracture injury in another specimen test. However, the physical device produces the same mechanical outcome for the same input regardless of the number of tests conducted on the physical device. Needless to state, structural outcomes are absent because the surrogate is infrangible by design. Data from the two surrogates are used in the substitution method to derive dummy-specific injury criteria.

### Simple Substitution Method Used for Dummy-Based Injury Criteria

In this method, the anatomical/structural outcome from the biological surrogate is matched with measured dummy metrics to derive dummy-specific injury assessment risk curves (IARCs) from which injury assessment risk values (IARVs) are extracted. For example, the metric corresponding to a predetermined risk level extracted from the logistic regression probability curve representing the IARC represents the IARV at that risk magnitude. The process involves duplicating the predictor response variable from the dummy-measured experiments for all tests conducted with the biological surrogate under the same input loading and boundary conditions. However, anatomical/structural outcomes (presence or absence of injury if the statistical model is binary) depend on the specimen. In other words, for the identical measured metric (for example, spine acceleration) from the dummy test under one initial condition, specimens sustaining injuries are coded as associated with yes injury, whereas specimens remaining intact after the test are coded as associated with no injury data points. Different statistical models can be used with this approach. This may include binary regression type, Weibull or logistic models, or survival analysis with different distributions. Covariates can also be used with these models. Biomechanical studies have used this substitution approach for developing dummy-based IARCs and IARVs (Kuppa et al., 2003; Cavanaugh and Yoganandan, 2015).

### Need for a New Infusion Methodology

It is well known that demographics factors of the biological surrogate play a role in the resulting injury and its severity. Thus, it is appropriate to include this variable and modulate the dummy output parameter, although the dummy is a physical device. This is also desirable to facilitate an appropriate age-specific injury probability curve for the dummy when a defined population is targeted for a specific application. The above described, currently used, substitution approach does not account for such influencing variables because the same magnitude of the predictor variable from the dummy test is used for different specimen tests with varying injury outcomes for a given initial condition to derive IARCs. A more appropriate approach would be to incorporate injury influencing factors, such as age, into the derivation of IARCs. This perspective article proposes a process, termed the age-infusion methodology, to modulate the dummy predictor variable based on the age of each matched-pair tested biological surrogate for the derivation of IARCs and extraction of IARVs. The feasibility and generalizability of the methodology are demonstrated using matched-pair side-impact sled test data from PMHS and ES-2re dummy.

## Age-Infusion Methodology

### PMHS and Dummy Experiments

Previously reported side-impact tests using unembalmed, intact PMHSs are used to demonstrate the age-infused methodology (Pintar et al., 1997; Maltese et al., 2002; Kuppa et al., 2003). Briefly, the specimens were dressed in tight-fitting leotards, placed on a Teflon-coated bench seat fixed to the platform of a sled and configured with an impacting load wall to simulate a side impact. The upper, middle, and lower segmented plates were used for contacting the thorax, abdomen, and pelvis, and a lower extremity plate was used in the segmented wall design. The configuration of the wall was such that it was flat or incorporated an initial pelvic or thoracic lead. The Frankfort plane of the PMHS was horizontal, legs were parallel to the mid-sagittal plane, and normal curvature and alignment of the dorsal spine were maintained before applying the side-impact pulse. The end conditions on the load wall were padded, rigid, or initial pelvic/thoracic lead. Each PMHS was palpated following the sled test, a clinical-type examination was performed, x-rays were obtained, and a detailed autopsy was conducted. Resulting injuries to the hard and soft tissues were graded using the 1990–1998 update of the abbreviated injury scale (AIS, 1990). A parallel process with the exception of biological details was used for the ES-2re dummy sled tests. Tests were done under each input and end condition used in the PMHS series to obtain dummy-based responses. The response data averaged from the dummy tests under the same input pulse and end condition were matched with the PMHS data to develop the IARCs and IARVs, as described below.

### PMHS Probability Curves

Probability curves were developed using the resultant spinal acceleration as the primary explanatory variable and age as the secondary variable (covariate). Simple logistic regression analysis was used for the statistical analysis. The human cadaver injury probability curve (HCIPC) was derived for the mean age of the tested PMHS ensemble, termed the mean curve. Then, this mean curve was converted to a risk curve representing 40 years of age, termed the reference age. The change in the response at the reference age was obtained by normalizing the ratio of the resultant spinal acceleration at the reference age (obtained from the HCIPC) with respect to the acceleration at the mean age of the ensemble (obtained from the mean curve). The annual rate factor was determined by normalizing the ratio with the difference between the reference and mean ages, using the following equation.

(1) $$\text{Annual rate factor}=\left(P_{\text{ref}}∕P_{\text{mean}}\right)∕\text{mod}{\text{(Age}}_{\text{ref}}-{\text{Age}}_{\text{mean}}\text{)}$$

*P*_ref_ represents the acceleration at reference age, and *P*_mean_ represents the acceleration of the corresponding to the mean age of the PMHS ensemble, both obtained using the respective HCIPCs. Mod refers to modulus, and Age_ref_ and Age_mean_ refer to the reference age and mean age of the entire ensemble.

### Dummy Matched-Pair Tests and Risk Curves

Matched-pair tests were also conducted in the cited references with the ES-2re dummy under different input and end conditions used in PMHS tests described above. Spine accelerations from the dummy were averaged when more than two tests were conducted for each condition and used as the primary explanatory variable and age was used as the covariate. The dummy-based IARCs were derived using the data duplication or the simple substitution approach and age-infused methodology.

In the simple substitution approach, the dummy-based IARC was derived by substituting PMHS-measured accelerations with dummy-measured accelerations. The process involved duplicating the same peak magnitude of the dummy acceleration to each PMHS specimen tested under the “same” pulse and end condition, although injury outcomes depended on the individual specimen characteristic that included age as a demographic factor. The IARC was determined for the dummy reference age, selected as 40 years as stated above, and using the same logistic regression model. In the age-infusion methodology, the process involved the incorporation of each PMHS age regardless of input pulse and end condition to dummy-measured accelerations. Thus, the process resulted in changing the dummy-measured spine acceleration based on the individual specimen age. The following equation was used to determine the equivalent dummy acceleration for each PMHS specimen. This was termed the infused PMHS specimen-specific dummy acceleration corresponding to the reference age.

(2) $$\begin{array}{l} R_{\text{ref}-\text{i}}=R_{\text{dummy}-\text{i}}\times \left[1-\left(\text{Annual rate factor}\times \left({\text{Age}}_{\text{PMHS}-\text{i}}-{\text{Age}}_{\text{mean}}\right)\right)\right] \end{array}$$

*R*_ref−i_ represents the infused PMHS specimen-specific dummy acceleration corresponding to the ith PMHS specimen, *R*_dummy−i_ represents the measured response of the dummy from the matched-pair condition, i.e., same input pulse and end condition, and Age_PMHS−i_ refers to age of the ith PMHS specimen for which the dummy acceleration is matched. In other words, the dummy acceleration for the same input pulse and end condition corresponding to the ith PMHS specimen was altered based on the age of the ith PMHS specimen and annual rate factor determined from Eq. 1. This dataset, i.e., infused PMHS specimen-specific dummy acceleration to the ith PMHS specimen and age and injury outcomes of the ith PMHS specimen, was used to derive the IARC by following the same statistical model used for the derivation of HCIPCs. Results are compared between the simple substitution, i.e., data duplication, and current age-infusion methodologies. Specifically, IARCs and IARVs at different probability levels are compared to demonstrate the differences in the two approaches.

### Human Cadaver Injury Probability Data

**Figure 1:**
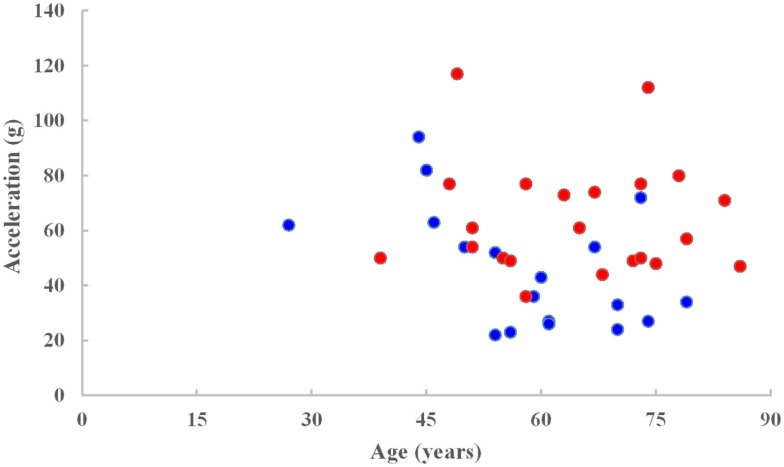
**Spine acceleration versus age for injury (red) and no injury (blue) data points**.

The PMHS ensemble consisted of 42 intact sled tests with 10 high speed and 28 low speed (8.9 and 6.7 m/s) input pulses. The mean age of the entire ensemble was 62 ± 13 years, range: 27–86 years. The end conditions were such that there were 14 padded and 24 rigid wall tests, 8 offset, and 4 airbag tests. The injury outcome was dichotomized between AIS < 3 (sample size = 17) and AIS ≥ 3 (sample size 23) levels. Mean peak acceleration was 56 ± 23 *g* and ranged from 22 to 117 *g*. Peak accelerations of 24 and 50 (IRV) were associated with the 20 and 50% probability of thoracic injuries based on the logistic regression model for the mean age of the ensemble and 49 and 74 *g* at the same risk levels for the reference age. Figure 1 shows acceleration versus age plot for injury and no injury outcomes.

### Dummy IARC and IARV Outcomes

**Figure 2:**
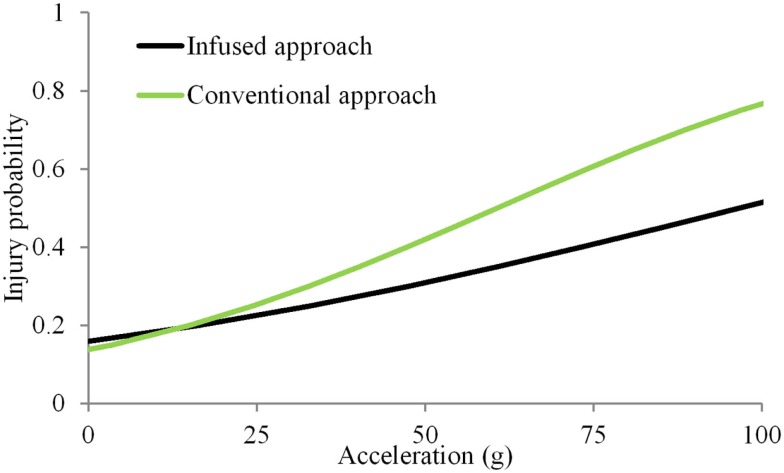
**Injury probability curves using the simple substitution and infused approaches**. The simple substitution curve is based on duplicating acceleration magnitudes for all specimens tested under the same initial condition regardless of injury outcomes (Kuppa et al., 2003). The age-infused curve is based on modifying the dummy-measured acceleration based on the individual specimen age.

In the simple substitution approach, the dummy dataset consisted of duplicating accelerations for nine subsets of different combinations of velocity, use of energy absorbing material and load wall geometry. In addition, different sample sizes were available under each subset. Mean peak accelerations ranged from 22 to 93 *g* (38 ± 19 *g*). Accelerations of 15 and 61 *g* (IARV) were associated with the 20 and 50% probability of injuries (Figure 2). Magnitudes of accelerations >100 *g* were considered to be beyond the bounds of the expected results from automotive tests. Therefore, the IARCs from both methods are plotted up to this limit. In the infusion approach, the dummy response data were scaled up or down to accommodate this annual rate based on the age of each PMHS to obtain the spinal acceleration for the infused dataset. Mean peak accelerations ranged from 11 to 150 *g* (40 ± 28 *g*). Results from these infused magnitudes along with corresponding matched-pair PMHS age and injury outcomes indicated that accelerations of 16 and 97 *g* were associated with the 25 and 50% probability of injuries.

## Comments on the Age-Infusion Methodology

The currently used simple substitution approach involves duplicating the same magnitude of the peak acceleration for the dummy dataset for each PMHS experiment conducted under similar input and end conditions (sled pulse and padded or rigid load wall with the same geometrical configuration for the wall). In other words, if there are seven PMHS specimens in a group, the peak dummy acceleration magnitude is the same for all seven specimens although PMHS-based accelerations are different. This is because the dummy produces the same (within its repeatability) magnitude of acceleration for a specific input pulse and end condition. The duplication process results in a cluster of data points. This process affects risk curves and accelerations at pre-selected injury probability levels. The age-infusion methodology presented induces a spread of dummy accelerations by introducing the age modifier on a specimen-by-specimen basis.

The infusion approach was based on the fact that injury outcomes (fracture and no fracture, or fractures of varying severity) and biomechanical metrics (such as accelerations) of biological materials depend on demographics, especially age. This is true for bones, such as the human lumbar vertebrae, and femur and its neck wherein bone mineral density is inversely proportional to age regardless of the sex variable and the method of measurement of the mineral content, quantitated computed tomography or densitometry (Hansson and Roos, 1980; Riggs et al., 1981; Yoganandan et al., 1988, 2006). Bone mineral data continue to be used in the assessment of osteopenia and osteoporosis in clinical settings around the world to predict fracture risk. It is also true in biomechanics wherein age is shown to significantly influence the strength of the human spine and its components under different loading conditions (Pintar et al., 1998). Furthermore, age affects impact responses (peak acceleration, deflection, etc.) of the human body and its components, as shown in other PMHS studies (Kuppa et al., 2001). Therefore, it is necessary to accommodate this important variable not only in the determination of human tolerance via HCIPCs but also in the development of IARCs and IARVs because the latter two statistical measures are used to advance crashworthiness and improve occupant safety in motor vehicle environments. The infusion approach proposed in this research with age to modulate the dummy response for each PMHS specimen, based on this biomechanical premise, yields more appropriate outcomes.

The age-infusion process to modulate the response variable involves the determination of the annual rate factor. Defined based on the response of the entire PMHS ensemble using HCIPCs for the mean and reference ages, this factor relies both on biomechanical and statistical outcomes. The mean ratio of accelerations was computed across the 15–85% probability levels to demonstrate the feasibility of the age-infusion approach. This range was chosen to accommodate a wide range in the human cadaver curves. However, other risk levels and ranges can be used to determine the rate factor. The dummy reference age can also be altered based on the type of application, for example, military scenarios are biased to lower age than the automotive group. The proposed infusion approach is flexible to accommodate such features.

The two approaches initiated approximately at the same magnitude of acceleration at a risk level close to 0. The non-zero response for a null stimulus is incongruent with mechanical induced injury and is due to the selection of the statistical model. Other models may be used in conjunction with the logistic regression to more accurately represent the response due to mechanical forces (Nakahira et al., 2000). However, such methods have not received wide attention. While the analysis chosen is in line with numerous biomechanical studies, other models can be used with the infusion approach (Rupp et al., 2010; Arun et al., 2015; Rupp, 2015). At other risk levels, differences existed between the age-infusion and simple substitution approaches, with the infusion methodology demonstrating increasing variations with increasing probability ranges. The injury risk curve from the infusion methodology resulted in a right and down-shifted curve compared to the simple substitution approach. The increased accelerations with increasing risk levels indicate that the age-infusion process considerably alters the response. The level of change or increase stems from the PMHS ensemble, its response output and the age selected for the dummy. Wider variations in the ensemble demographics contribute to this result as other factors besides age, such as body mass index, may influence the injury metric.

The infusion methodology incorporated the chosen reference age for the dummy, individual ages of each tested PMHS specimen and HCIPCs to derive an annual rate factor to modulate dummy responses for the determination of IARC and IARV. The feasibility of using the infusion approach was demonstrated using a set of PMHS accelerations and injury outcomes and matched-pair dummy accelerations. The dataset used in this article was meant to demonstrate the feasibility of the infusion methodology. Other parameters, such as force and deflection, can also be used as explanatory variables. The example chosen used logistic regression. Other models ranging from simple binary logistic regression to more detailed survival analysis can be used with this methodology. The quality of the PMHS-derived risk curves is a function of the dataset and measures, such as log-likelihood ratio. Modern safety-related advancements are targeted at lower injury risks. For example, lower leg injury criterion is specified at 10% risk level in the military and the trend is along similar lines in motor vehicle-related crashworthiness research (NATO, 2007; Prasad et al., 2010; Yoganandan et al., 2014a, 2015a). Because of stated biomechanical rationale, the infusion approach accounting for the age variable better describes the underlying biomechanical phenomena at different probability levels.

## Conclusion

The current framework of using age in the risk function can only be adopted when the statistical analysis shows that the age is significant. If it is not, it is statistically inappropriate to calculate the injury risk curve for a specific dummy age. The proposed concept parallels PMHS dataset of uniqueness of both age and response by modifying ATD-measured responses based on the rate factor derived from the PMHS dataset. Because the modification is based on the injury risk curve from PMHS (which includes age and response to injury) for the ATD-specific age, there is no need to reintroduce age into the regression equation to derive the risk curve when the statistical model shows that age is an insignificant covariate.

Substitution methods for injury risk assessments limit the use of age as a covariate. Dummy metrics (e.g., accelerations) are not modified, so that they can be directly included in the model. A methodology is described in this perspective article for modifying the dummy injury risk assessment responses to account for the age of the PMHS that the injury data were based on. Specifically, the age-based infusion approach to modify dummy injury risk curves and injury criteria will serve as an improvement to current methods of determining injury criteria in which age is not accounted in this manner for the derivations of IARCs and IARVs (Kuppa et al., 2003). This may also provide future directions to guide PMHS testing and to develop crashworthiness standards as applied to automotive and other environments, wherein dummies are used to assess human safety.

## Other Considerations and Limitations

It should be noted that not all human injury curves and assessment values are developed with direct applications to dummies. For example, applications include injury assessments in falls and certain types of sports activities (Hayes et al., 2007). However, safety in automotive, aviation, and military generally rely on the application of human cadaver data to ATD and as stated, the present study is focused on such applications. The infusion methodology calls for modulating the dummy metrics based on the individual age of the PMHS and then using a statistical model to derive the risk curve. The presented example used logistic regression model, which shows a finite ordinate at the origin of the risk curve (Figure 2). However, age-modulated data can be used to derive risk curves using models, such as Weibull distribution and survival models. If the target population/reference age falls beyond the age range used in PMHS experiments (28–86 years of age used in the dataset), one can extrapolate linearly to obtain the factor. However, risk curves derived from data extrapolated beyond the experimental range should be considered as preliminary. This process underscores the need to obtain data from specimens that include the targeted reference age for the dummy. Because of the perspective type of the article, detailed review of literature is not possible. The reader is referred to additional papers on injury risk curves (Eppinger et al., 1999; Laituri et al., 2006; Prasad et al., 2010; Rupp et al., 2010; McMurry and Poplin, 2015) and age dependency on mechanical properties of tissues (Bartley et al., 1966; Melick and Miller, 1966; Lindahl and Lindgren, 1967; Yamada, 1970; Burstein et al., 1976; Currey, 1979; Hansson and Roos, 1980; Riggs et al., 1981; McCalden et al., 1993; Zhou et al., 1996; Pintar et al., 1998). The cited list in not all inclusive.

## Ethics Statement

This study used published data. No experiments were conducted for this perspective article.

## Conflict of Interest Statement

The authors declare that the research was conducted in the absence of any commercial or financial relationships that could be construed as a potential conflict of interest.
